# Exploration of spatial distribution of brain metastasis from small cell lung cancer and identification of metastatic risk level of brain regions: a multicenter, retrospective study

**DOI:** 10.1186/s40644-021-00410-w

**Published:** 2021-06-13

**Authors:** Yong Wang, Wei Xia, Baoyan Liu, Liu Zhou, Meng Ni, Rui Zhang, Jingyi Shen, Yujun Bai, Guixiang Weng, Shuanghu Yuan, Xin Gao

**Affiliations:** 1 Cheeloo College of Medicine, Shandong University, Jinan, Shandong China; 2 Shandong Cancer Hospital and Institute, Shandong First Medical University and Shandong Academy of Medical Sciences, No.440, Jiyan Road, Jinan, 250117 Shandong China; 3 Suzhou Institute of Biomedical Engineering and Technology, Chinese Academy of Sciences, 88 Keling Road, Suzhou New District, Suzhou, 215163 Jiangsu China; 4 Jinan Guoke Medical Engineering and Technology Development Co., Ltd., Pharmaceutical Valley New Drug Creation Platform, Jinan, Shandong China; 5 Shandong Academy of Occupational Health and Occupational Medicine, Shandong First Medical University & Shandong Academy of Medical Sciences, Jinan, Shandong China; 6 Jihua Laboratory, Foshan, Guangdong China; 7 Fudan University Shanghai Cancer Center; Shanghai Medical College, Fudan University, shanghai, China; 8 Linyi People’s Hospital, Linyi, China

**Keywords:** Whole brain radiation therapy, Brain metastases, Small-cell lung cancer, Location analysis

## Abstract

**Objectives:**

This study aimed to explore the spatial distribution of brain metastases (BMs) from small cell lung cancer (SCLC) a homogenous sample, and to identify the metastatic risk levels in brain regions.

**Methods:**

T1-enhanced magnetic resonance imaging (MRI) from SCLC patients were retrospectively reviewed from three medical institutions in China. All images were registered to the standard brain template provided by the Montreal Neurological Institute (MNI) 152 database, followed by transformation of the location of all BMs to the space of standard brain. The MNI structural atlas and Anatomical Automatic Labeling (AAL) atlas were then used to identify the anatomical brain regions, and the observed and expected rates of BMs were compared using 2-tailed proportional hypothesis testing. The locations and sizes of brain lesions were analyzed after image standardization.

**Results:**

A total of 215 eligible patients with 1033 lesions were screened by MRI, including 157 (73%) males and 58 (27%) females. The incidence of crucial structures were as follows: hippocampus 0.68%, parahippocampal 0.97%, brainstem 2.05%, cauate 0.68%, putamen 0.68%, pallidum 0.2%, thalamus 1.36%. No BMs were found in the amygdala, pituitary gland, or pineal gland. The cumulative frequency of the important structures was 6.62%. Based on the results of MNI structural atlas, the cerebellum, deep white matter and brainstem was identified as a higher risk region than expected for BMs (*P* = 9.80 ×10^−15^, 9.04 ×10^−6^), whereas temporal lobe were low-risk regions (*P* = 1.65 ×10^−4^). More detailed AAL atlas revealed that the low-risk regions for BMs was inferior frontal gyrus (*P* = 6.971 ×10^−4^), while the high-risk regions for BMs was cerebellar hemispheres (*P* = 1.177 ×10^−9^).

**Conclusion:**

Many crucial structures including the hippocampus, parahippocampus, pituitary gland and thalamus etc. have low frequency of brain metastases in a population of SCLC patients. This study provides the help to investigate the clinical feasibility of HA-WBRT and non-uniform dose of PCI in a population of SCLC patients.

**Supplementary Information:**

The online version contains supplementary material available at 10.1186/s40644-021-00410-w.

## Background

Small cell lung cancer (SCLC) is one of the most common malignancies worldwide, with a prevalence of 13 to 15% among all types of lung cancer. Over 25% of SCLC patient were initially diagnosed with brain metastases (BMs), with an average survival of 9 months after maximal treatment [1–3]. Yet as the imaging techniques and systemic therapies develop, so does the enhanced incidence of BMs year by year due to prolonged survival of patients. The presence of BMs remains a significant contributor to overall cancer mortality in patients at an advanced-stage [4]. Prophylactic cranial irradiation (PCI) has been widely applied to prevent the development of BMs for SCLC patients [5, 6]. Despite strong evidence supporting PCI for SCLC, there is an increased risk of neurotoxicity, which may cause cognitive decline [7]. To reduce the neurological toxicity caused by cranial irradiation, Gondi et al. performed whole-brain radiation therapy (WBRT) with hippocampus sparing (HS) in patients with multiple BMs using radiation oncology working group (RTOG) 0933 trial, and the results showed that a reduction of adverse neurocognitive effects with HS WBRT compared with WBRT alone. In this study, SCLC or germ cell malignancy was excluded due to the concern for a higher rate of failure in the hippocampus [8].. The NRG CC001 study, a phase III trial that replicated the results of the RTOG0933 study, showed that the use of hippocampal avoidance during WBRT with memantine effectively spares the hippocampal neuro-regenerative niche to better preserve cognitive function and patient-reported symptoms, and no differences were observed in toxicity, intracranial PFS, or OS compared with standard WBRT and memantine. However, the primary tumors included in this study were mixed. Although the primary tumors were matched between groups, the detailed information of each primary tumor, such as the number of SCLC patients and the number of BMs, was not revealed [9]. To further explore the role of hippocampal protection in patients with limited and extensive stage SCLC receiving PCI, a Phase 2/3 randomized trial of NRG CC003 is currently underway. The primary endpoint of this clinical trial is to determine whether hippocampal protected PCI reduces the likelihood of neurocognitive deterioration at 6 months after treatment. A recent analysis within the EORTC 22033 clinical trial reported that the hippocampus normal tissue complication probability model did not perform as expected to predict cognitive decline based on dose to 40% of the bilateral hippocampus [10]. Perhaps more important structures also need to be protected, and a risk-adapted WBRT method for the SCLC patients is worthy of further investigation.

Image analysis with CT or MRI has become the typical approach for the relationship between the spatial distribution of BMs and primary lung cancer types. The development of image registration and 3D structure deformation algorithms allow the detection of the slightest differences in spatial distribution by registering individual brain images onto an averaged standard brain image. Accumulating evidences have suggested that the spatial distribution of BMs from different types of cancer are different [11, 12]. Quattrocchi et al. [11] have highlighted the non-uniform spatial distribution of BMs in patients with breast and lung cancer patient by using MRI scans. One recent study has demonstrated that several critical brain structures have a low risk of developing BMs, however, only 19 cases (6.9%) of BMs were from SCLC [13]. Yet, there is still uncertainty about results. The unique characteristic of small SCLC may play a significant role in distant metastasis. Histological subtype and mutation status have become increasingly important in BMs from cancer, especially non-small cell lung cancer (NSCLC) [14]. However, no discussion or analysis of BMs from SCLC regarding to its spatial distribution has been reported.

In this study, we analyzed the spatial distribution of BMs from SCLC in a large database from three medical institutions in China. Two different atlases were also applied to identify the location of BMs in different brain regions, as well as the risk level of metastasis in the brain.

## Materials and methods

### Patients and data collection

This study was approved by each institutional review board, and each ethics committee waiver informed consent due to the character of this research. A total of 215 SCLC patients with BMs from January 2014 to December 2018 were enrolled in this research from three medical institutions in China. Inclusion criteria were as follows: (1) patients firstly diagnosed with BMs from SCLC and had no identification of BMs in the previous MRI; (2) no contradictions to MRI; (3) To reduce the divergence in the identification of the BMs for different clinicians, the patients with obvious metastases which had the number of metastases ≤20, and metastasis diameter ≥ 2 mm were included. Exclusion criteria were: (1) patients with history of brain surgery or any form of brain radiation therapy; (2) patients with meningeal metastasis; (3) the presence of other types of malignant tumors.

#### MRI protocol

Coil: 8-channel cross cranial coil. Position: supine position. T1WI, T2WI, and T2WI-FLAIR of the head were followed by enhanced scanning. Scanning parameters: T1WI TR: 250 ms, TE: 2.3 ms, layer thickness: 6 mm, number of excitation: 1; T2WI TR: 2105 ms, TE: 80 ms, layer thickness: 6 mm, number of excitation: 1. Contrast-enhanced scanning: Gd-DTPA was injected intravenous mass and measured at 0.1 mmol /kg. Transaxial, sagittal and coronal T1WI scanning was performed, respectively.

### Spatial distribution analysis of BMs

The volume of interest (VOI) of each BM on MRI was manually delineated slice-by-slice using Medical Imaging Toolkit (MITK) software (version 2013.12.0; http://www.mitk.org/). Image registration was performed to transform the coordinate space of MRI image data to the standard Montreal Neurological Institute (MNI) space. The high-resolution T1 weighted standard brain template with 1.0 mm isotropic provided by the MNI152 database was adopted as the reference image [15], and each volume of MRI scan was registered to the standard brain template with a 12 degree of freedom transformation using the Linear Imaging Registration Tool of FSL library (FSL-FLIRT, version 6.0; https://fsl.fmrib.ox.ac.uk/fsl) on Ubuntu 16.04 [16].

In the affine registration, the cost function is the correlation ratio with trilinear interpolation and the size of 256 bin. A transformation matrix which aligned with each volume of MRI scan to the standard brain template was generated after image registration. The VOIs were transformed to the standard MNI space by applying the registration derived transformation matrix with nearest neighborhood interpolation using the FSL-FLIRT. The centroid of each VOI was calculated using Matlab software (version R2017a) and recorded as the location of BM.

Both MNI structural atlas [15] and an more detailed Anatomical Automatic Labeling (AAL) atlas [17] were used to identify anatomical brain regions in the standard brain template. The MNI structural atlas segmented the brain to 9 regions. The AAL atlas segmented the brain as 116 cortical, subcortical, and cerebellar regions. To focus on the important brain regions, the segmented brain regions were merged into 38 regions. Since some important structures were not annotated in atlas, the pituitary gland, pineal gland, brainstem, cerebellar vermis and cerebellar hemisphere were manually delineated in MNI standard brain template using MITK software. For each brain region, the number of BMs in the whole cohort was counted.

To visualize the spatial distribution of BMs, a frequency map was constructed as binary spheres with a diameter of 20 mm at the locations of BMs and some spheres may be overlaid. A heat-map for the overlap frequency of all spheres was counted at each voxel [14].

### Statistical analysis

In each anatomical brain region, the observed rate of BMs was calculated as the number of BMs in the region divided by the number of BMs in all regions. By assuming an equal risk of metastasis from SCLC for each voxel, the expected rate of BMs in each brain region was calculated as the proportion of region’s volume in the total volume of all regions. To identify the risk of metastasis in each brain region, the observed and expected rate of BMs were compared using 2-tailed proportional hypothesis testing [18] as follows:

$$ \mathrm{Z}=\frac{p-{p}_0}{\sqrt{\frac{p_0\left(1-{p}_0\right)}{n}}} $$

Where, *p* is the observed rate of BMs, *p*_0_ is the expected rate of BMs, *n* is the number of BMs, and Z is the z-score.

The corresponding *P* value of the z-score was calculated as the cumulative probability of normal distribution, and the Bonferroni correction was used for the correction of multiple testing across all anatomic ROIs. Therefore, the corrected significance level of *P* value was calculated as the significance level 0.05 divided by 53 which was the number of ROIs, and the *P* value needed to reach statistical significance was *P* ≤ 9.434 × 10–4. In the brain regions with statistically significant *P* values, the brain regions with positive or negative z-score were the regions which had observed rate of BMs significantly higher or lower than the expected rate of BMs.

## Results

### Patient characteristics

From the database of newly diagnosed patients with BMs from three medical institutions in China, a total of 215 eligible patients with 1033 lesions were screened, including 157 (73%) males and 58 (27%) females. The age was ranged between 36 and 83 years old, with a median age of 61 years. With regard to the location of the primary lung cancer, we observed 50.2% (108/215) of cases from the left lung as compared with a 49.8% (107/215) from the right lung. The time from the first diagnosis of SCLC to the onset of BMs was ranged from 0 to 45.5 months, with the median of 7.1 months. The median number of metastatic brain tumors was 5 per patient. Detailed patient characteristics and demographic information were shown in Table 1.

**Table 1:** Patient characteristics

characteristic	NO. of Patients(%)
Age (years)	
Median	61
Range	36–83
Gender	
Male	157 (73%)
Female	58 (27%)
Location of primary SCLC	
Left lung	108 (50.2%)
Right lung	107 (49.8%)
Time from SCLC to BMs (months)	
Median	7.1
Range	0–45.5
Lesion per patient	
Median	5
Range	1–18
Total lesions	1033

### Visualization of the spatial distribution of BMs and quantitative analysis of the observed incidence

**Fig. 1:**
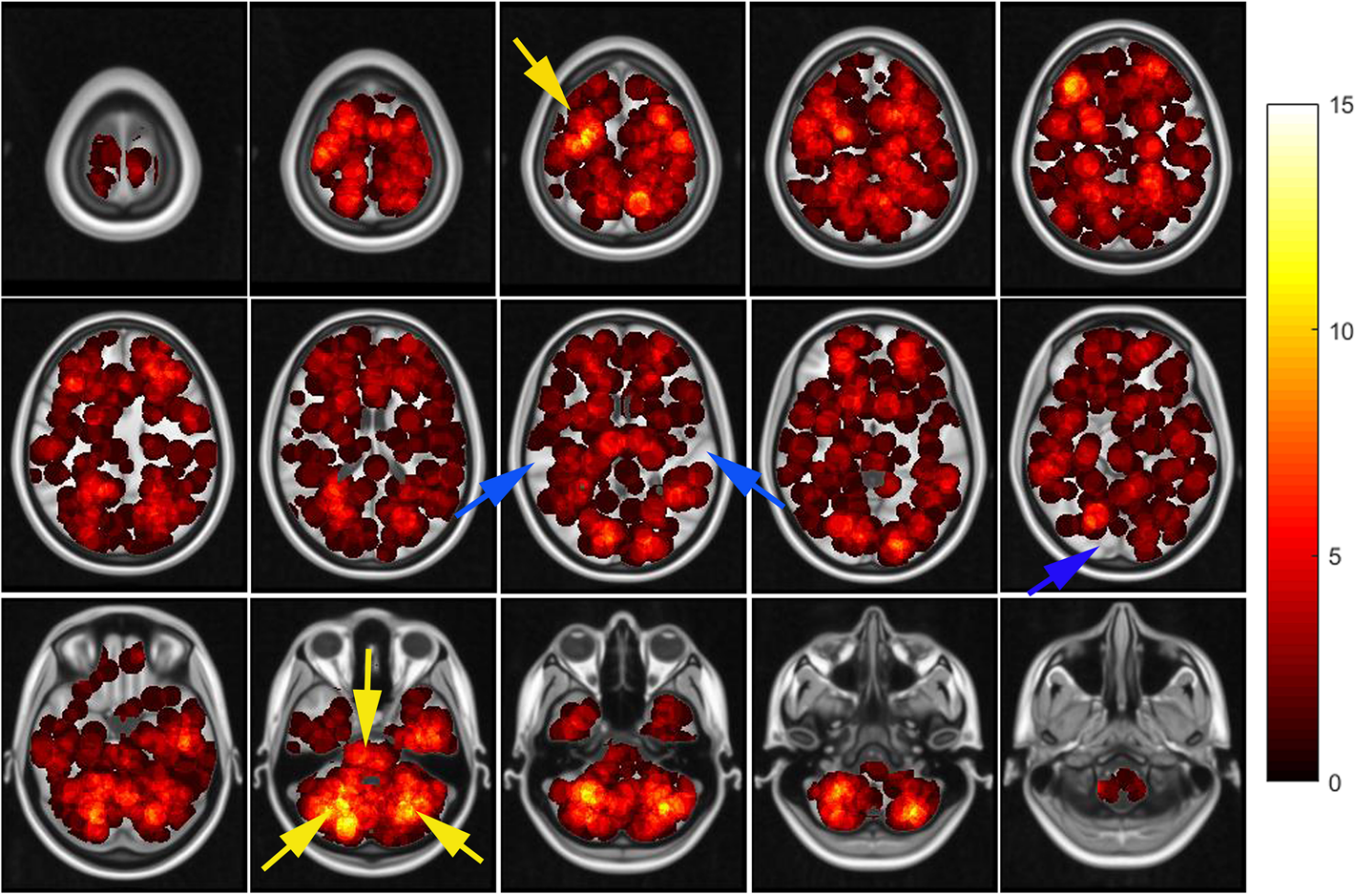
The axial images of the frequency map of all brain metastases from SCLC. From the heat map, we can intuitively feel that the brightness of each brain region is not uniform. The blue arrow indicates a significantly lower incidence, while the yellow arrow indicates a higher incidence

A heat map of the spatial distribution of BMs was obtained by superimposing the normalized tumor centroid (Fig. 1). Visualized heat maps show that the brightness of different regions of brain function is not uniform. Frontal lobe regions, bilateral cerebellum and brainstem regions were highlighted, indicating a high incidence of BMs; however, the brightness of bilateral temporal lobe and part of occipital lobe were low, indicating a low incidence of BMs. We used MNI152 atlas (9 regions) and merged AAL atlas (38 regions) to achieve automatic segmentation of brain functional areas, and quantitatively analyzed the number and incidence of BMs in each brain regions. In the brain regions segmented by MNI152 atlas (Table 2), the BMs frequency were as follows: frontal lobe 27.0% (277), cerebellum 20.9% (214), parietal lobe 16.6% (170), temporal lobe 11.2% (115), deep white matter and brainstem 10.5% (108), occipital lobe 8.2% (84), caudata 1.8% (18), thalamus 1.6% (16), insula 1.2% (12), putamen1.2% (12). In the brain regions segmented by merged AAL atlas, the six areas of top BMs frequency were as follows: cerebellar hemispheres 9.84% (101), middle frontal gyrus 6.71% (69), gyrus frontalis superior 6.34% (65), cerebellar peduncle 5.85% (60), precentral gyrus 4.48%, (46), cingulate gyrus 3.03% (31). Meanwhile, many crucial regions had a very low frequency, such as hippocampus 0.68%, parahippocampal 0.97%, brainstem 2.05%, cauate 0.68%, putamen 0.68%, pallidum 0.2%, thalamus 1.36%. No BMs were found in the amygdala, pituitary gland, or pineal gland. The cumulative frequency of the above important structures was 6.62%. The incidence of deep white matter metastasis was 10%. The specific BMs occurrence and frequency data are collated together in Table 3, and supplementary Table.

**Table 2:** Results from the analysis based on MNI structural atlas

Region	Volume, mm^3^	Expected Rate (%)	No. Observed	Observed Rate (%)	z-score	*P*-Value
Caudate	32,353	1.4	18	1.8	0.953	0.341
Cerebellum	294,552	12.8	214	20.9	7.745	9.80 × 10^-15^
Frontal lobe	708,087	30.7	277	27.0	−2.593	0.010
Insula	33,041	1.4	12	1.2	−0.713	0.476
Occipital lobe	236,954	10.3	84	8.2	−2.212	0.027
Parietal lobe	421,103	18.3	170	16.6	−1.415	0.157
Putamen	26,171	1.1	12	1.2	0.102	0.919
Temporal lobe	356,227	15.5	115	11.2	−3.768	1.65 × 10^-4^
Thalamus	34,802	1.5	16	1.6	0.129	0.898
Deep white matter and brainstem	161,082	7.0	108	10.5	4.440	9.04 × 10^-6^

**Table 3:** Statistically significant results from the analysis based on AAL atlas

Region	Volume, mm^3^	Expected Rate (%)	No. Observed	Observed Rate (%)	z-Score	*p*-Value
hippocampus	15,024	0.79	7	0.68	−0.39	0.699
parahippocampus	16,880	0.89	10	0.97	0.30	0.763
brainstem	40,087	2.11	21	2.05	−0.13	0.896
caudatum	15,648	0.82	7	0.68	−0.49	0.621
putamen	16,584	0.87	7	0.68	−0.65	0.516
pallidum	4584	0.24	2	0.2	−0.30	0.765
pituitary gland	1088	0.06	0	0	−0.785	0.444
pineal gland	404	0.02	0	0	−0.467	0.641
amygdala	3744	0.19	0	0	−1.422	0.155
precentral gyrus	55,256	2.9	46	4.48	3.02	0.003
inferior frontal gyrus	84,112	4.42	23	2.24	−3.39	6.971 ×10^−4^
cingulate gyrus	61,240	3.22	31	3.03	−0.35	0.724
occipital gyrus (superior, middle, inferior)	80,616	4.23	31	3.0	−1.93	0.054
supramarginal gyrus	25,840	1.36	5	0.49	−2.41	0.016
temporal pole	36,520	1.92	12	1.17	−1.75	0.081
Heschl’s gyrus	3792	0.19	3	0.29	0.67	0.503
gyrus temporalis superior	43,496	2.28	10	0.97	−2.81	0.005
gyrus temporalis medius	74,808	3.93	29	2.82	−1.82	0.069
gyrus temporalis inferior	54,056	2.83	21	2.05	−1.53	0.127
cerebellar hemispheres	104,904	5.51	101	9.84	6.09	1.177 ×10^−9^
vermis cerebelli	16,320	0.86	10	0.98	0.41	0.683
cerebellar peduncle	74,096	3.89	60	5.85	3.24	0.001

### Identification of the metastatic risk level by comparing the observed frequency with the expected frequency

Based on the MNI atlas, the comparison of the observed and expected rates of BMs showed that the cerebellum, deep white matter and brainstem were area with higher risk than expected (*P* = 9.80 ×10^−15^, 9.04 ×10^−6^), whereas temporal lobe, frontal lobe and occipital lobe were regions with lower risk than expected (*P* < 0.05). The temporal lobe remained significant after the correction of multiple testing (*P* = 1.65 ×10^−4^). Analysis based on a more detailed ALL atlas shows that BMs incidence was lower than expected in many deep brain structures, including inferior frontal gyrus (2.24% VS 4.42%), superior temporal gyrus (0.97% VS 2.28%), supramarginal gyrus (0.49% VS 1.36%), rectus gyrus (0.1% VS 0.67%), occipital superior-middle-inferior gyrus (3% VS 4.23%), middle temporal gyrus (2.82% VS 3.93%), temporal pole (1.17% VS 1.92%), inferior temporal gyrus (2.05% VS 2.83%), cuneus gyrus (0.78% VS 1.24%), angular gyrus (0.78% VS 1.23%), hippocampus (0.68% VS 0.79%) etc. From Table 3, the frequency of observed BMs was significantly different from the expected value in 7 ROIs (*P* < 0.05), including cerebellar hemisphere, inferior frontal gyrus, cerebellar peduncle, precentral gyrus, superior temporal gyrus, supramarginal gyrus, rectus gyrus. The BMs lower risk than expected areas were inferior frontal gyrus, superior temporal gyrus, supramarginal gyrus, rectus gyrus. In contrast, the remaining regions have higher risk than expected. The cerebellar hemispheres and inferior frontal gyrus remained significant after the correction of multiple testing (*P* = 1.177 ×10^−9^, 6.971 ×10^−4^).

**Fig. 2:**
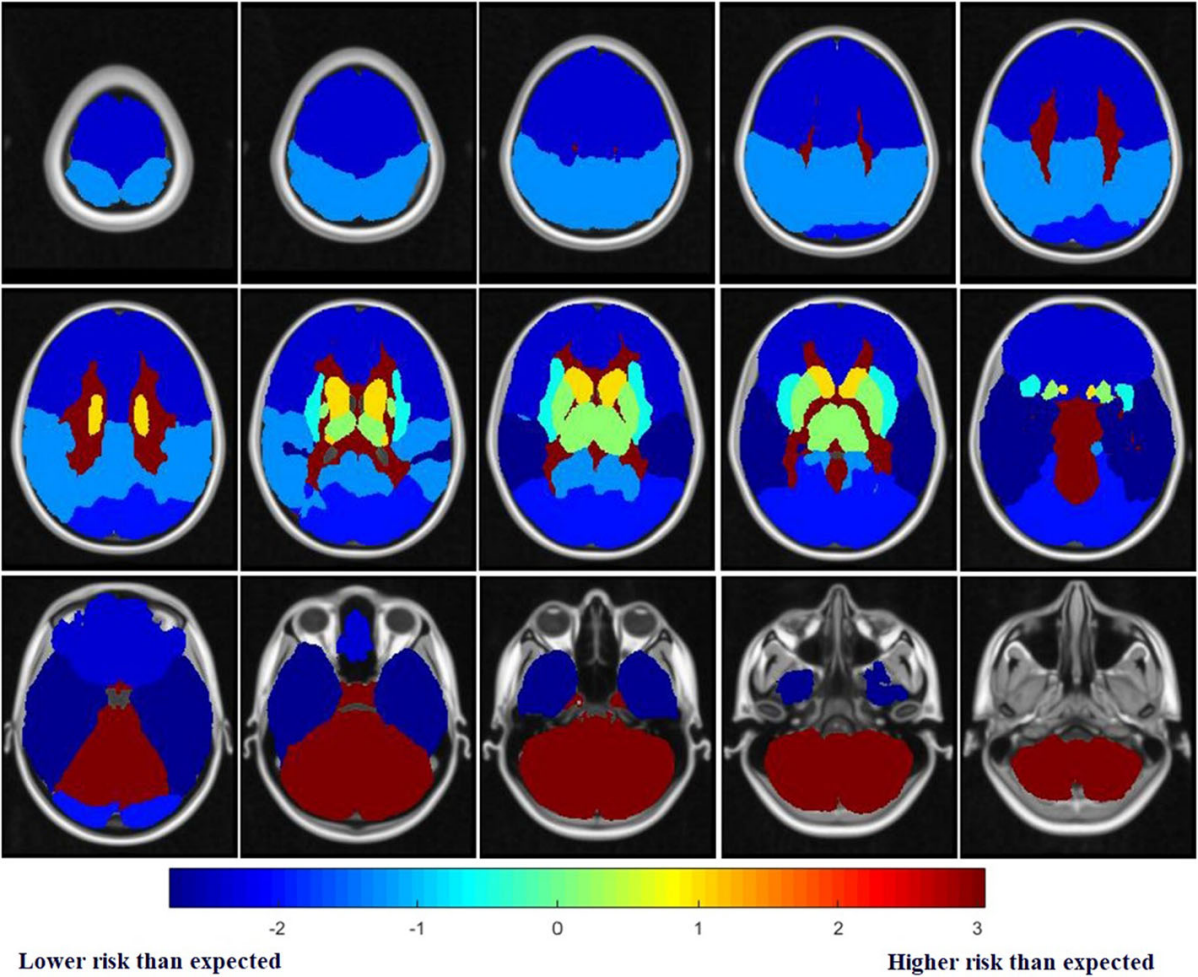
Risk level visual axial heat map imaging based on MNI152 atlas. Red: high z-score and risk level. Blue: In contrast, low z-score and risk level

**Fig. 3:**
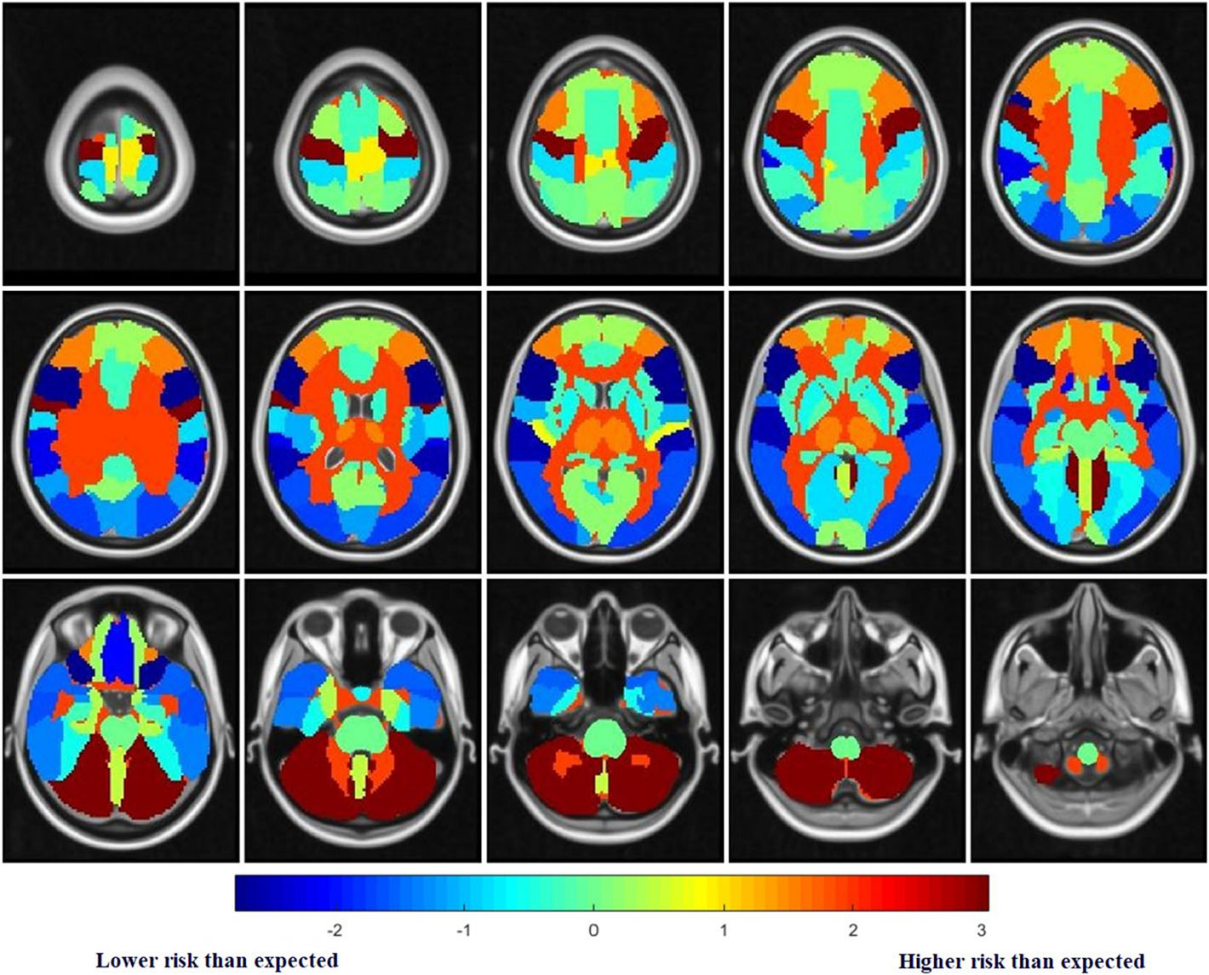
Risk level visual axial heat map imaging based on ALL atlas. Red: high z-score and risk level. Blue: In contrast, low z-score and risk level

The risk was visualized according to *P* value and Z-score. The heatmap represents the risk level of lesions in brain regions. Red represents higher Z-scores and higher risk level, whereas blue represents lower Z-scores and lower risk level. The risk level visualization based on MNI atlas is shown in Fig. 2, and the risk level visualization based on ALL atlas is shown in Fig. 3.

## Discussion

The spatial distribution of BMs varies in different types of cancer. Previous study has confirmed that the spatial distribution of BMs in lung cancer patients is different from that in breast cancer patients [11]. NSCLC patients are more likely to develop BMs in the parietal-occipital lobe and cerebellum region, while the cerebellar hemisphere is the potential location of breast cancer BMs. In addition, Takano K [14] has further suggested that the spatial distribution of BMs from NSCLC and SCLC is different even though they are lung cancers. Compared to NSCLC, SCLC has small tumor cells with the biological characteristics of early metastasis, more diffuse distribution of BMs and deeper BMs [19–22]. The BMs from SCLC had the worst prognosis among all the BMs of lung cancer. PCI is identified as one of the major treatments for SCLC [6]; however, its preventive effect is not optimal due to high recurrence rate and neurotoxicity [5, 23–25]. Several clinical studies have believed that hippocampus-sparing radiation avoids the neurotoxic side effects to some extent [8, 26–28]. NRG CC001, a phase 3 trial based on the RTOG0933 study, showed that HA-WBRT plus memantine better preserves cognitive function and patient-reported symptoms, with no difference in intracranial PFS and OS [9]. This has spurred interest in research into hippocampal conservation. However, patients with SCLC have generally been excluded from these randomized clinical trials, so the use of HA-WBRT remains controversial in small cell lung cancer. NRG-CC003 is a separate clinical trial designed to answer questions about hippocampal protection in patients with SCLC. Prior to the completion of the NRG-CC003 trial, it is necessary to study the spatial distribution of BMs in small cell lung cancer alone. And we believe that the findings of this study could make important observations about this.

In this study, we hypothesized that the spatial frequency of BMs from SCLC is non-stochastic distribution, and that the risk of metastasis varies across brain regions. To test this, we identified 1026 BMs in 215 patients from three medical institutions, and both MNI152 atlas (10 regions) and ALL atlas (38 regions) to determine the anatomical brain regions in standard brain template, then compared observed rates of BMs to the expected rates of BMs under the assumption that all voxels were at equal risk. The frequency heat map of BMs from SCLC showed that the BMs from SCLC were mainly located at the frontal lobe (27%) and cerebellum (20.9%), while both insula and putamen regions (1.2%) had the lowest frequency of BMs. More detailed quantitative analysis data are presented in tables for future inquiry by other researchers.

In our study, it was found that only 0.68% of the metastases were located in the hippocampus and 0.97% in the parahippocampus. Such results were similar to those reported by Wu et al. [29]. by 0.5% in the hippocampus and 0.6% in the peri-hippocampal metastasis. However, they were slightly higher than reported (0.68% VS 0.5, 0.97% VS 0.6%), which may be related to the fact that we are a homogeneous single group of SCLC. Our study found that the incidence of hippocampal metastasis in SCLC is still less than 1%, so we believe that HA-WBRT is safe in this situation. In addition, we found that the incidence of metastases to the hippocampus and parahippocampal gyrus was only 1.65%, so it seems acceptable to relax the strict boundary delineation of the hippocampus when limited by radiotherapy equipment.

In addition to protecting the hippocampus and neural stem cells, it is debatable whether other important functional structures such as the limbic system, amygdala, hypothalamus, pineal gland, pituitary gland, and areas near the inner ear need to be protected as well [30]. The limbic system is involved in regulating instinctual and emotional behavior through associations with the hypothalamus and the vegetative nervous system. The hypothalamus is the highest center under the vegetal cortex, an important connection point between the limbic system and the reticular structure, and the stimulation site of the pituitary endocrine system. Although small in size, the hypothalamus receives a large number of nerve impulses and is the center of the endocrine and nervous systems. Metastases in the hypothalamus can lead to abnormalities in motivational behavior, such as feeding, drinking, sexual behavior, fighting, thermoregulation, and activity levels. The amygdala is an emotional structure in the brain that recognizes emotions and regulates them, controlling learning and memory. Both pituitary gland and pineal gland are important endocrine structures in brain. Protective radiotherapy is bound to increase the risk of recurrent brain metastases in protected areas, which requires a detailed analysis of the risk of brain metastases in these areas [31]. Our study found a 5.26% incidence of key structures, including the hippocampus, parahippocampus, brainstem, caudatum, putamen, pallidus, thalamus, pituitary gland and pineal gland. Based on this, the risk of recurrence associated with protecting these areas appears to be acceptable. However, this is controversial and difficult because it is already challenging to cover brain with sufficient dose while sparing hippocampus. These problems need to be solved by future advances in radiotherapy technology.

To further verify the non-random spatial distribution of BMs in SCLC, we assumed that each voxel had the same risk of SCLC metastasis. The expected rate of BMS in each brain area was calculated as a proportion of brain area volume to total brain volume. Through comparison, we found that the observation frequency of cerebellum was significantly higher than the expected frequency (*P* = 9.80 ×10^−15^), while the observation frequency of temporal lobe, frontal lobe and occipital lobe was significantly lower than the expected frequency, and the difference of temporal lobe was more significant (*P* = 1.65 ×10^−4^), which was similar to the results of previous studies on heterogenous primary pathological type of brain metastases [11, 13, 32]. In order to make the research more thorough, more detailed atlas was applied to the segmentation. The frequency of observations of precentral gyrus, cerebellar hemispheres, and cerebellar peduncle regions was significantly higher than expected. While the observation frequency of the inferior frontal gyru, supramarginal gyrus, gyrus temporalis superior, and gyrus rectus were significantly lower than their expected frequency. Even after corrected multiple tests, there were still significant differences in the areas of inferior frontal gyrus (*P* = 6.97 ×10^−4^) and cerebellar hemispheres (*P* = 1.17 ×10^−9^). Our new findings fully validate the non-random spatial distribution of BMs in SCLC.

Identification of metastatic risk level of brain regions in this study could help the modern treatment techniques to achieve risk-adapted PCI, such as intensity-modulated radiation therapy (IMRT) and volumetric modulated arc therapy (VMAT), allowing the delivery of high dose radiation to the high-risk brain regions, and low dose radiation to the low-risk regions [13]. This modification may improve the efficacy of PCI and reduce the neurotoxicity of PCI in SCLC patients with BMs.

Limitations of our study should also be noted. First, no brain metastases were found in several brain regions, which may be due to the low brain volume in these regions, or the small sample size, which needs further study to confirm. Second, since our study only focused on BMs from SCLC, the differences in spatial distribution between SCLC and other pathological BMs need to be further compared, which is precisely what we lack. Both of these limitations may be solved by further analysis with a larger cohort to reach definite conclusions.

## Conclusions

We confirmed that the cerebellum was identified as a higher risk region than expected for BMs from SCLC, whereas temporal lobe was a lower risk region than expected. More detailed atlas revealed that the cumulative BMs incidence of many important structures was low in a population of SCLC patients, including the hippocampus, parahippocampus, pituitary gland, basal ganglia, thalamus, and so on. This study provides the help to investigate the clinical feasibility of HA-WBRT and non-uniform dose of PCI in a population of SCLC patients.

## Supplementary Information

**Additional file 1: Supplementary Table 1.** Distribution of brain metastases based on AAL atlas.

## Data Availability

The datasets used and/or analyzed during the current study are available from the corresponding author on reasonable request.

## Abbreviations

BMs: Brain metastases
SCLC: Small Cell Lung Cancer
MNI: Montreal Neurological Institute
AAL: Anatomical Automatic Labeling
PCI: Prophylactic Cranial Irradiation
WBRT: Whole-brain Radiation Therapy
HS: Hippocampus Sparing
RTOG: Radiation Oncology Working Group
CT: Computed tomography
MRI: Magnetic Resonance Imaging
EGFR: Epidermal Growth Factor Receptor
NSCLC: Non-small cell lung cancer
VOI: Volume of interest
MITK: Medical Imaging Toolkit
IMRT: Intensity-modulated radiation therapy
VMAT: Volumetric modulated arc therapy
